# Supplementary material for: Reconciling Mining with the Conservation of Cave Biodiversity: A Quantitative Baseline to Help Establish Conservation Priorities
Source: PLoS One. 2016 Dec 20;11(12):e0168348. doi: 10.1371/journal.pone.0168348 (PMC5173368; doi:10.1371/journal.pone.0168348)
Supplement: S1 Dataset — (ZIP) [file pone.0168348.s002.zip › Taxa/Serra Norte/SN_2013/N4E/Lista N4E-03.pdf]

CAVIDADE N4E-0003

Classe	Ordem	Fam/Outros	Gên/Outros	Espécie	S-U	U-S	Zon.
Annelida	Oligochaeta			jovem		1	P
Arachnida	Acari	(Oribatida)		jovem	X		
Arachnida	Acari	Cunaxidae		sp.3		X	A
Arachnida	Acari	Neothyridae	*Diplothyrus*	sp.1		X	P
Arachnida	Acari	Prostigmata		jovem	X		
Arachnida	Amblypygi	Phrynidae	*Heterophrynus*	*longicornis*	X		
Arachnida	Araneae	Corinnidae		jovem		2	A
Arachnida	Araneae	Ochyroceratidae	*Ochyrocera*	sp.3	X	X	P/A
Arachnida	Araneae	Ochyroceratidae	*Speocera*	sp.	X		
Arachnida	Araneae	Prodidomidae		jovem		X	P
Arachnida	Araneae	Theridiidae		sp.	X		
Arachnida	Araneae	Theridiosomatidae	*Plato*	sp.1	X	X	A
Arachnida	Opiliones	Escadabiidae		sp.2		X	P
Arachnida	Palpigradi	Eukoeneniidae	*Allokoenenia*	sp.1		X	A
Arachnida	Pseudoscorpiones	Bochicidae		sp.	X		
Arachnida	Pseudoscorpiones	Chernetidae		sp.	X		
Arachnida	Pseudoscorpiones	Chthoniidae	*Pseudochthonius*	sp.1	X	X	P
Arachnida	Ricinulei	Ricinoididae	*Cryptocellus*	jovem		1	P
Arachnida	Schizomida	Hubbardiidae	aff. *Rowlandius*	sp.1	X	X	P
Chilopoda	Scolopendromorpha	Scolopocryptopidae	*Newportia*	*ernsti*	X		
Diplopoda	Glomeridesmida	Glomeridesmidae		sp.1	X	2	P
Diplopoda	Polydesmida	Chelodesmidae		sp.	X	3	P
Diplopoda	Polydesmida	Fuhrmannodesmidae		jovem		X	A
Diplopoda	Polydesmida	Pyrgodesmidae		sp.1	X	X	A
Diplopoda	Siphonophorida	Siphonophoridae		jovem		1	A
Entognatha	Collembola	Isotomidae		sp.1		X	P
Entognatha	Collembola	Paronellidae		sp.1		X	P
Entognatha	Collembola	Paronellidae		sp.4		X	A
Entognatha	Collembola	Sminthuroidea		sp.2		X	P
Entognatha	Diplura	Campodeidae		sp.1	X	X	P
Entognatha	Diplura	Japygidae		sp.1		X	P
Insecta	Blattodea			jovem	X		
Insecta	Coleoptera	Lampyridae		jovem	X		
Insecta	Coleoptera	Ptiliidae		jovem		X	P
Insecta	Coleoptera	Staphylinidae		sp.52		X	P
Insecta	Diptera	Drosophilidae	*Drosophila*	sp.	X		
Insecta	Diptera	Psychodidae	Phlebotominae	sp.		X	P/A
Insecta	Diptera	Psychodidae	*Pintomyia*	*gruta*	X		
Insecta	Diptera	Psychodidae	*Sciopemyia*	*sordellii*	X		
Insecta	Diptera	Tipulidae		sp.	X		
Insecta	Heteroptera	Cydnidae		sp.	X		
Insecta	Homoptera	Cixiidae		jovem	X	X	P
Insecta	Hymenoptera	Formicidae	*Hypoponera*	sp.	X		
Insecta	Hymenoptera	Formicidae	*Odontomachus*	*haematodus*	X		
Insecta	Hymenoptera	Formicidae	*Odontomachus*	sp.3		X	P
Insecta	Hymenoptera	Formicidae	*Pachycondyla*	*constricta*	X		
Insecta	Hymenoptera	Formicidae	*Paratrechina*	sp.	X		
Insecta	Lepidoptera	Tineoidea		jovem	X		
Insecta	Orthoptera	Phalangopsidae	*Phalangopsis*	sp.	X	1	P
Gastropoda	Pulmonata	Bulimulidae	*Naesiotus*	sp.		X	P/A
Gastropoda	Pulmonata	Subulinidae	*Lamellaxis*	sp.	X		
Gastropoda	Pulmonata	Subulinidae	*Leptinaria*	sp.	X		
Gastropoda	Pulmonata	Systrophiidae	*Happia*	sp.	X	X	P/A
Malacostraca	Isopoda	Philosciidae		sp.1	X	X	P
Malacostraca	Isopoda	Scleropactidae	*Circoniscus*	*buckupi*	X		

Nematoda				sp.1		X	A
Symphyla		Scutigerellidae	*Hanseniella*	sp.	X		
Turbellaria	Tricladida			sp.1		1	P
Mammalia	Chiroptera	Phyllostomidae	*Carollia*	*perspicillata*		1	A
